# Altered network efficiency in major depressive disorder

**DOI:** 10.1186/s12888-016-1053-9

**Published:** 2016-12-17

**Authors:** Ming Ye, Peng Qing, Ke Zhang, Guangyuan Liu

**Affiliations:** 1 College of Computer and Information Science, Southwest University, Chongqing, 400715 China; 2 College of Electronic and Information Engineering, Southwest University, Chongqing, 400715 China; 3 School of Computer Science, Sichuan University of Science and Engineering, Sichuan Zigong, 643000 China

**Keywords:** Network topology, Network efficiency, Major depressive disorder, Functional magnetic resonance imaging, Cognitive control system

## Abstract

**Background:**

Major depressive disorder (MDD) is associated with dysfunction between cognitive control and affective processing system. However, little is known about alterations of the nodal and edge efficiency in abnormal systems of MDD patients. We used two independent datasets and two different structural templates to investigate the alterations of the nodal and edge efficiency of whole-brain functional networks of MDD.

**Method:**

Forty-two MDD and forty-two age, education-matched controls were selected to investigate network efficiency abnormalities of the MDD patients’ cortical and subcortical regions, as well as the disrupted functional connectivity between these regions, from the perspective of network topological architectures. In addition, another dataset, which included thirty MDD patients and thirty controls, was also investigated using the same method.

**Results:**

Results showed that MDD group demonstrated significant increase in the local efficiency, although not change of global efficiency. In addition, nodal efficiency was found to increase in affective processing regions (i.e., amygdale, thalamus, hippocampus), but decrease in cognitive control related regions, which included dorsolateral prefrontal cortex and anterior cingulate cortex. The edge efficiency was found to increase, involving both connectivity between thalamus and limbic system regions and connectivity between hippocampus and regions (i.e., amygdala, thalamus). More important, result was replicated within independent datasets for the first and different structural templates for another.

**Conclusions:**

Our results indicated that MDD was associated with disrupted functional connectivity networks between cognitive control and affective processing systems. The findings might shed light on the pathological mechanism of depression and provide potential biomarkers for clinic treatment of depression.

**Electronic supplementary material:**

The online version of this article (doi:10.1186/s12888-016-1053-9) contains supplementary material, which is available to authorized users.

## Background

Major depressive disorder (MDD) is one of world’s most prevalent psychiatric disorders [14], which is characterized by inappropriate symptoms [6], such as losing interest in daily activities, psychomotor retardation, sleep disturbance, exacerbating the experience of negative effects and even attempt to suicide. Moreover, about 30 % patients with MDD do not respond to standard antidepressant treatment [21] and patients who recovered still have 80 % probability cure other psychiatric disorders of relapse [14].

Substantial structural and functional neuroimaging researches on MDD revealed a complex neuropathophysiology involving regional deficits in the prefrontal-thalamo-limbic and limbic-striatal-pallidal-thalamic systems [21]. The dysfunctional systems mainly involved cortical regions (mainly part of cognitive control network), such as dorsolateral prefrontal cortex (DLPFC) [12, 14] and anterior cingulate cortex (ACC) [9] and subcortical regions (mainly part of affective processing network), which included amygdala [28], hippocampus [22], parahippocampal gyrus [23], caudate nucleus [26], posterior cingulate cortex [23] and thalamus [25]. As for the cognitive control network, previous studies found that depressed patients showed impaired attentional disengagement from negative stimuli, which need top-down regulation and executive functioning from cortical regions such as the DLPFC [7, 12]. In addition, ACC, which was associated with inhibition, would contribute to impaired disengagement [7]. Prior studies demonstrated that MDD would have greater activation when successfully inhibiting attention to negative stimuli [10, 11], while normal controls would demonstrated greater rostral ACC activity when successfully inhibiting attention to positive stimuli. These results suggested that MDD patients would need more cognitive effort to divert attention away from negative stimuli [7]. On the other hand, affective processing network, including subcortical regions such as amygdala, thalamus, demonstrated diverse functional activity patterns. It is well-known that emotional stimuli would be projected to the amygdala through the transfer of thalamus (LeDoux). Then, amygdala, a brain structure that is involved in detecting emotion, would interpret and perpetuate the emotional quality of the stimulus. This process would be regulated in part by indirect inhibitory input from the DLPFC [8]. When depressed patients process negative stimuli, amygdala would show greater reactivity and longer lasting [8, 29], which may be associated with DLPFC aberrant activation. As with the activation pattern of amygdala in individuals of depression, hippocampus would show enhanced activity in recall of negative, not positive stimuli [14], after encoding in the amygdala.

Thus, it is of necessity to investigate functional connectivity networks in the whole brain of the depression. In addition, it would be useful for identification of diagnosis biomarkers [4, 31] for MDD patient and advanced our understanding of the neuropsychopathology of depression to some extent. Previous studies suggested that depression is also associated with topological disorganization of brain networks, including disrupted global integrity and regional connectivity [18, 20, 32]. However, recent review suggested that previous studies which examined the topological properties of brain functional networks with depression contained only one group of subjects and the results could not be duplicated. In addition, the structural template was one important influence factor for topological properties. However, previous studies [20, 32] mainly investigate the topological properties within one structural template. Thus, independent datasets and the same analysis strategy within different structural template were important and needed to be investigated.

In the present study, we constructed brain function networks of two independent datasets by using resting-state functional magnetic resonance imaging (rs-fMRI). Here, nodes are defined by anatomical regions of AAL template and Harvard-Oxford Atlases respectively. Connectivity between regions was defined as edges. We calculated correlations between cortical and subcortical regions to measure functional connectivity matrices. Then, the correlation matrices were thresholded to construct brain functional networks. We finally analyzed the topological properties of brain functional connectivity networks and compared the differences between patients with MDD and controls. Based on the previous studies, we hypothesized that MDD patients would show dysfunction in regions of cognitive control network (e.g., DLPFC and ACC) and affective processing network (e.g., amygdala, thalamus, hippocampus and parahippocampal gyrus). In addition, the altered functional connectivity networks would be observed within regions of the resting-state networks which were associated with affective and cognitive control processing [7].

## Methods

### Subjects

Two independent datasets was randomly selected from our ongoing project, which examined the occurrence and development of depression. In the first dataset, 98 subjects (49 MDD and 49 controls), whose age from 18 to 60 years participated in the experiments. In the second dataset, another 36 MDD and 35 control was selected into the present study. Inclusion criteria for MDD subjects are: (1) patients meet the judgment of MDD defined by DSM-IV, which was diagnosed by experienced psychiatrists from the First Affiliated Hospital of Chongqing Medical University and with score of Hamilton Depression Rating Scale (HAM-D) larger than 24, (2) no history of major medical or neurological abnormalities (e.g.: head trauma with loss of consciousness, migraine, cyst, or unusually large ventricles); (3) not have metallic implants or other factors which will influence fMRI examination; (4) diagnosed as depressive disorder, but not bipolar disorder; (5) no presence of alcohol or substance abuse. Inclusion criteria for all healthy subjects are: (1) no drug abuse and alcohol abuse in past two weeks, no drug dependence and alcohol dependence in past one year, (2) no history of psychiatric or neurological disorder and no significant family history of psychiatric or neurological disorder.

This study was approved by the ethics committee of School of Psychology at Southwest University, China. We had obtained appropriate ethics committee approval for the research reported, and all subjects have gave written informed consent.

### Image acquisition

The experiments are processed in the Southwest University, Chongqing, China, using Siemens Trio 3-T scanner (Siemens, German Erlangen). All subjects were required to close their eyes and relax themselves without falling asleep. Subjects laid supine with their head fixed by belt and foam pads to reduce head movement. Thirty-two transaxial gradient echo planar imaging was acquired using the echo planar imaging sequecnce (thickness/gap = 3/1 mm, matrix = 64*64, repetition time (TR) = 2000 ms, echo time (TE) = 30 ms, flip angle = 90°,field of view = 220 mm × 220 mm). A total of 242 volumes were acquired in this scan for each subject.

### Functional data preprocessing

The preprocessing of functional images for the two datasets were independently carried out using the SPM8. The first 10 volumes were removed to allow for signal equilibration. Then, the remaining 232 volumes which we obtain from each subject were realigned to the first volume of head-motion correction in consideration of the acquisition time delay among different slices. In order to spatially normalized to the EPI template, the fMRI images were resampled to 3-mm cubic voxel. To compensate for residual within-subject variability, proceeding images were smoothed using a isotropic Gaussian filter (full-width at half-maximum = 6-mm). This step decreased high spatial frequency noise and made sure that the gaussian random field theory would obtain more applicability on further statistical testing. Then, we removed the several spurious variance sources such as 6 motion parameters, linear drift and the average time series in the cerebrospinal fluid and white matter regions from for each subject. Eventually, temporal band-pass filtering (0.01 ~ 0.08 Hz) was adjusted to reduce the low-frequency drift and high-frequency effect.

### Functional connectivity matrix and graph construction

For each dataset, two different structural templates were both calculated. First was anatomical automatic labeling (AAL) template; the brain is divided into 90 anatomical regions of interest and there exist 45 regions in each hemisphere. Another template was Harvard-Oxford Atlases; the brain was divided into 96 cortical regions and 16 subcortical regions. In order to analyze the functional connectivity among regions, there were many steps to follow. We first computed the average of the time series of all voxels in each region. Then a multiple linear regression model was used to remove the several resource of variance of BOLD signal from the mean time series. The estimated profiles of head motion and the global brain activity were repressors [13]. Then, we used the residual of regression as substitution for the corresponding regions’ raw mean time series. Finally, Pearson’s correlation coefficients were computed to produce the symmetric correlation matrix for each subject. Then, the matrix was transformed using Fourier Z-Transform. In order to gain the functional connectivity network, each Fourier Z-Transform matrix is threshold into binary graph [19], where regions as defined as nodes and connectivity between regions as undirected unweighted edges [5].

### Efficiency of the small-world networks

Achard [1] found that brain functional networks own small-world properties by investigating the efficiency and cost of human brain functional networks. In the present study, network cost was adopted because it coupled with network efficiency and provides description of the network’s performance. Here, the cost of network *G* = (*V*, *E*) which owns |*V*| nodes and |*E*| edges, measured how expensive to build a network and was defined in (1)

1 $$ C(G)=\frac{K}{N\left(N-1\right)/2} $$

where *K* = |*V*| and *N* = |*E*|. The denominator in eq.(1) means the number of all possible edges in network *G*. The cost threshold *C*(*G*) in a network determines the topology of network. High cost threshold yielded sparser network but low cost thresholded yield denser network. However, there was no accurate way to choose a threshold in the studies on brain networks [3, 15]. In order to analyze the difference of network properties in two groups, we investigated the properties of network over a wide range of cost threshold from 0.03 to 0.5. In the following analysis, in order to reduce the influence of cost on results, we investigated the network with different value of cost.

By given a cost threshold *C*(*G*), we could quantify properties of brain networks of MDD and control group using efficiency measure. The global efficiency of a network *G* was defined as eq.(2) [16, 17].

2 $$ {E}_{global}(G)=\frac{1}{N\left(N-1\right)}{\displaystyle \sum_{i\ne j\in G}\frac{1}{L_{i,j}}} $$

where *L*_*i*,*j*_ was the shortest length of the path from node *i* to node *j*. If *L*_*i*,*j*_ was infinite, it would make no contribution to the sum.

*E*_*global*_(*G*) measured the information propagation efficiency over network *G*. But *E*_*local*_(*G*) measured the local efficiency in network, which was defined in eq.(3) [16, 17] indicated how efficient the information exchanged in sub networks

3 $$ {E}_{local}(G)=\frac{1}{N}{\displaystyle \sum_{i\in G}E\left({G}_i\right)} $$

where *E*_*local*_(*G*) was the average of efficiency *E*(*G*_*i*_) of all sub network. *G*_*i*_ was a sub network in *G* and composed of nearest neighbors of node *i*.

Besides the above two metrics, we also defined the nodal efficiency, as in eq.(4), which measured the communication efficiency between node *i* and other nodes in network *G*

4 $$ {E}_{nodal}\left(G,i\right)=\frac{1}{N-1}{\displaystyle \sum_{j\in G}\frac{1}{L_{i,j}}} $$

Prior studies which applied efficiency to measure the brain functional networks ignored the edge efficiency between nodes. Here we used edge efficiency to investigate network properties, which could measure the contribution of edge *e* in the information propagation. The edge efficiency was defined in eq.(5)

5 $$ {E}_{edge}\left(G,e\right)=\frac{1}{L}{\displaystyle \sum_{i,j\in G,e\in {L}_{i,j}^{\prime }}\frac{1}{L_{i,j}^{\prime }}} $$

where *L*_*i*,*j*_^′^ was the shortest path from node *i* to node *j* passing through edge *e. L* was the number of shortest path in the network [33].

## Results

After excluding subjects with excessive motion (Dataset 1: 7 for MDD and 7 for control; Dataset 2: 6 for MDD and 5 for control), 42 MDD patients and 42 controls for dataset 1 (30 MDD patients and 30 controls for dataset 2) were included in our final analyses. Demographic and clinical characteristics for all subjects of the two datasets were in the Table 1. There were no significant differences between the groups with respect to age and education for the two datasets. As expected, the two groups differed significantly with respect to HAMD scores for the two datasets. The depression severity for all MDD patients reached severe level (24 or higher), with a mean HAMD score of 26.88 (SD = 2.92).

**Table 1:** Demographic and Clinical Characteristics of the Study Samples (two datasets^a^)

	MDD	Control	*t*	*p*	MDD	Control	*t*	*p*
Sample size (male)	42 (21)	42 (19)			30 (4)	30 (12)		
Age, mean (SD)	42.14 (12.33)	39.143 (11.72)	1.143	0.256	43.47 (15.71)	41.20 (10.96)	0.648	0.519
Education (year), mean (SD)	10.74 (3.90)	11.238 (3.43)	0.624	0.534	12.57 (3.60)	10.86 (3.40)	1.869	0.067
HAMD, mean (SD)	26.88 (2.92)	2.119 (1.783)	46.861	0.000	28.03 (3.74)	1.90 (1.81)	34.484	0.000
Medication-naïve	27^b^	NA			18	NA		
Past antidepressant use	14	NA			12	NA		
Duration of illness (month), mean (SD)	49.06 (68.10)^c^	NA			30.83 (41.77)^d^	NA		
Family history of psychiatric disorder^e^	4	NA			6	NA		
Comorbid generalized anxiety disorder	6	NA			11	NA		
Comorbid obsessive-compulsive disorder	1	NA			0	NA		

^a^Left for Dataset 1 and right for Dataset 2

^b^,^c^ and ^d^information for one subject was lost

^e^family history of depressive disorder up to second-degree relatives

HAMD, Hamilton Depression Scale; MDD, major depressive disorder; NA, not applicable

### Altered nodal efficiency in MDD patients

**Fig. 1:**
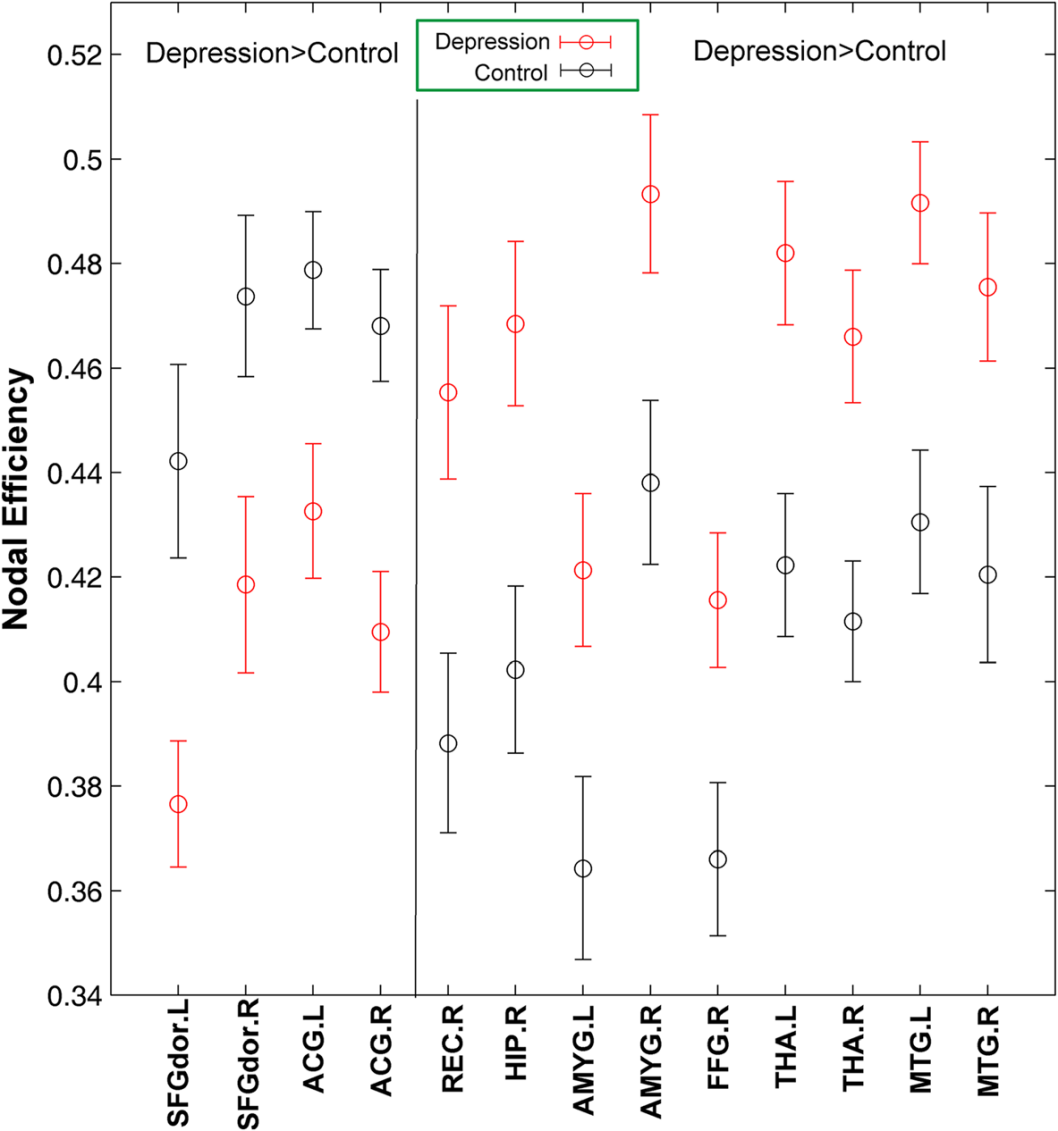
MDD-related changes in nodal efficiency when cost is 0.21. Red error bars correspond to the mean and standard error of the mean for MDD group; black error bars correspond to the mean and standard error of the mean for control group

**Fig. 2:**
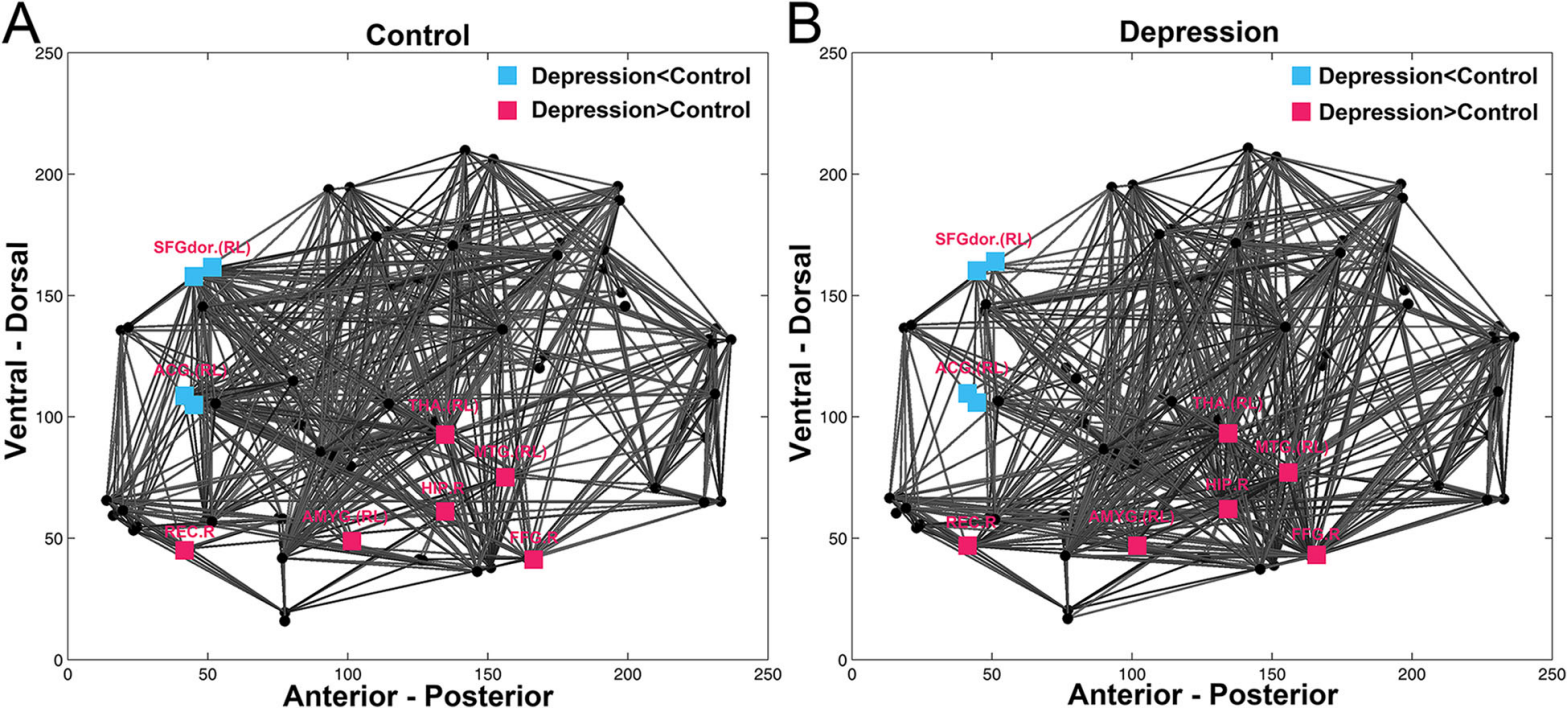
MDD-related changes in nodal efficiency in topological networks. **a**, **b** are left brain and right brain of the human. Networks are constructed by converting the individual correlation matrices at the cost of 0.21. Blue squares, red squares and black dots represent brain regions. Blue and red squares show that brain regions own lower and higher nodal efficiency in MDD subjects compared to controls, respectively

As the maximal difference was at the cost of 0.21 with AAL template, we tested the regionally nodal efficiency at the cost of 0.21 to reveal the aberrant nodal characteristics of the functional connectivity networks of MDD. As shown in Fig. 1 and Table 2, MDD patients demonstrated obvious increase in nodal efficiency in some regions (i.e., amygdala, thalamus, fusiform gyrus, hippocampus, rectus gyrus and middle frontal gyrus regions) and significant decrease in nodal efficiency was also found in regions in MDD (i.e., DLPFC and ACC). To further reveal the affected regions, we compared the brain topological networks of one control (see Fig. 2a) and one MDD subject (see Fig. 2b). Results with other value of cost and with another structural template were in the supplement 1. In addition, results could be replicated from the other dataset (see it in the Additional file 1).

**Table 2:** Changes of nodal efficiency at cost of 0.21 in MDD group when compared with controls (Dataset 1)

Regions	Hemisphere	*t* value	*P* value
Depression < Control			
Superior frontal gyrus,dorsolateral	L	−2.63	0.010
	R	−2.15	0.034
Anterior cingulate gyrus	L	−2.08	0.040
	R	−2.75	0.007
Depression > Control			
Gyrus rectus	R	2.56	0.012
Hippocampus	R	2.60	0.010
Amygdala	L	2.23	0.028
	R	2.20	0.030
Fusiform gyrus	R	2.10	0.039
Thalamus	L	2.52	0.013
	R	2.45	0.016
Middle temporal gyrus	L	2.68	0.009
	R	2.18	0.031

**Fig. 3:**
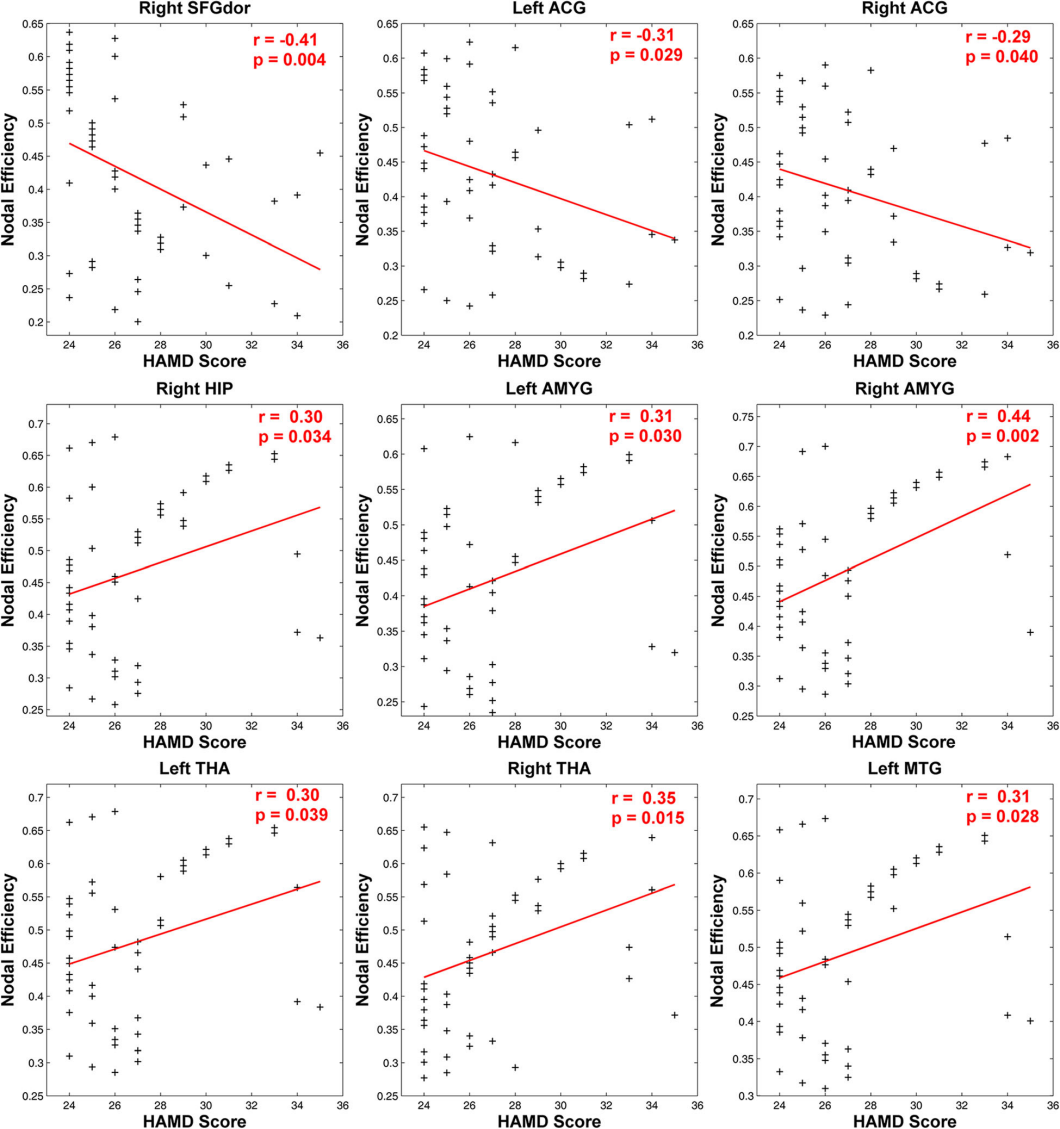
Upper scatter diagram shows correlation analysis between MDD nodal efficiency and HAMD Scores. The figure shows that there exist negative correlations between HAMD scores and nodal efficiency in regions (i.e., *right dorsolateral prefrontal cortex, bilateral anterior cingulate gyrus*), while positive correlation between HAMD scores and nodal efficiency in regions (i.e., *right hippocampus, bilateral amygdala, bilateral thalamus*)

Within the MDD group, we calculated pearson correlation coefficients to examine how nodal efficiency within the clusters relate to clinical severity. Results suggested that there were significant negative correlations between HAMD score and nodal efficiency of cognitive control regions (right dorsal superior frontal gyrus and bilateral anterior cingulate cortex). While HAMD score was found to positive correlate with several affective processing regions (right hippocampus, bilateral amygdala and thalamus) (see Fig. 3).

### Altered edge efficiency in MDD patients

**Fig. 4:**
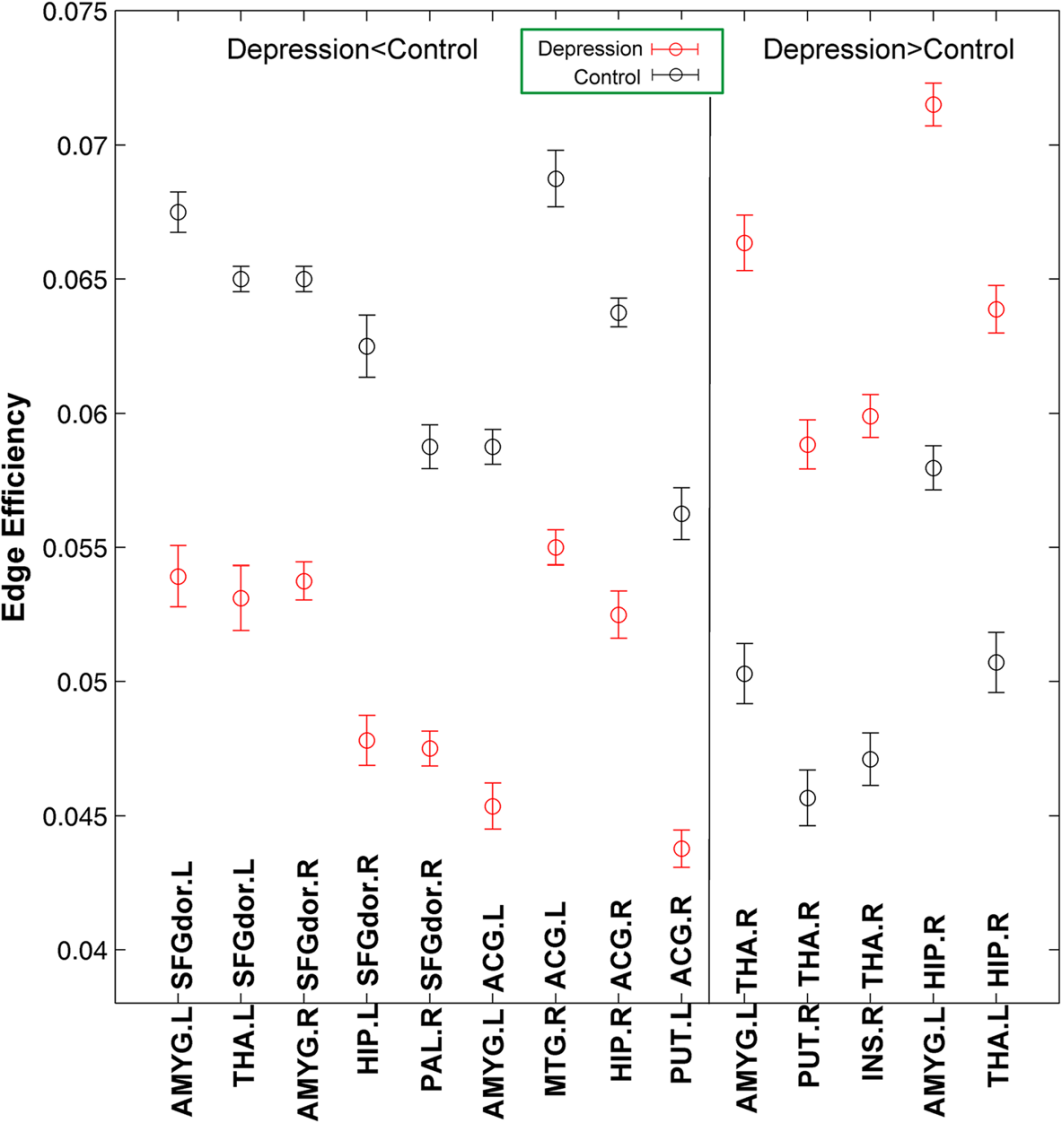
MDD-related changes in edge efficiency when cost is 0.21. Red error bars correspond to the mean and standard error of the mean for MDD group; black error bars correspond to the mean and standard error of the mean for control group

**Fig. 5:**
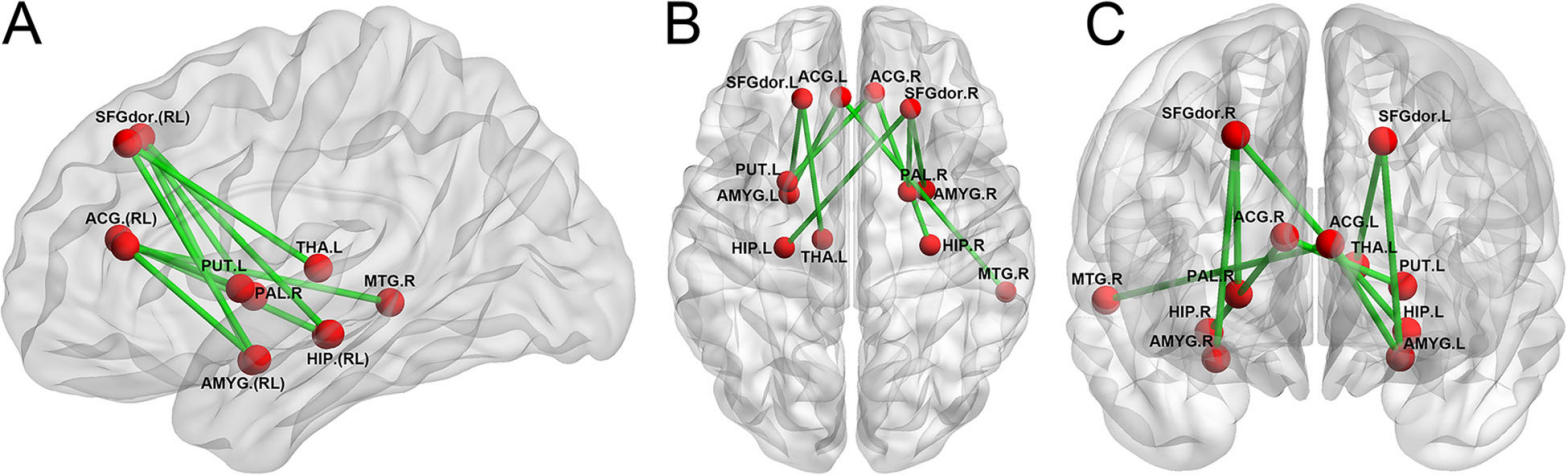
Three views of the location of edges whose efficiencies decrease in brain (refer to Table 2). Here, Red node represents regions, green line represents edge between regions. **a**, **b**, **c** are the side, top, back views respectively

As the nodal efficiency, correspond to the maximal between-group difference in the local efficiency, we further test the edge efficiency at the cost of 0.21 with AAL template. Results (as shown in Fig. 4 and Table 3) showed that obvious decrease in edge efficiency of MDD groups between DLPFC and regions (amygdala, thalamus, hippocampus, palladium), ACC and regions (amygdala, middle frontal gyrus, hippocampus, putamen). In addition, we also found significant increase in edge efficiency of MDD groups between thalamus and regions (amygdala, putamen, insula), between hippocampus and regions (amygdala, thalamus) (see it in Fig. 5). In addition, results with other value of cost and with another structural template were in the supplement 1. Moreover, results could be replicated from the other dataset (see it in the supplement 1).

**Table 3:** Results of edge efficiency when compared MDD group with control

Regions of seed	Connected regions	MNI Coordinates			*t* value	*p* value
		X	Y	Z		
Depression < Control						
Left Superior frontal gyrus, dorsolateral	Left Amygdala	−23	−0.7	−17	−2.18	0.031
	Left Thalamus	−11	−18	8	−2.03	0.045
Right Superior frontal gyrus, dorsolateral	Right Amygdala	27	0.6	−18	−2.30	0.023
	Left Hippocampus	−25	−21	−10	−2.25	0.027
	Right Lenticular nucleus, pallidum	21	0.2	0.2	−2.06	0.043
Left Anterior cingulate gyrus	Left Amygdala	−23	−0.7	−17	−2.42	0.018
	Right Middle temporal gyrus	57	−37	−1.5	−2.33	0.022
Right Anterior cingulate gyrus	Right Hippocampus	29	−20	−10	−2.09	0.039
	Left Lenticular nucleus, putamen	−24	3.9	2.4	−2.14	0.035
Depression > Control						
Right Thalamus	Left Amygdala	−23	−0.7	−17	2.42	0.018
	Right Putamen	28	4.9	2.5	2.09	0.039
	Right Insula	39	6.3	2.1	2.12	0.036
Left Hippocampus	Left Amygdala	−23	−0.7	−17	2.36	0.020
	Left Thalamus	−11	−18	8	2.06	0.042

**Fig. 6:**
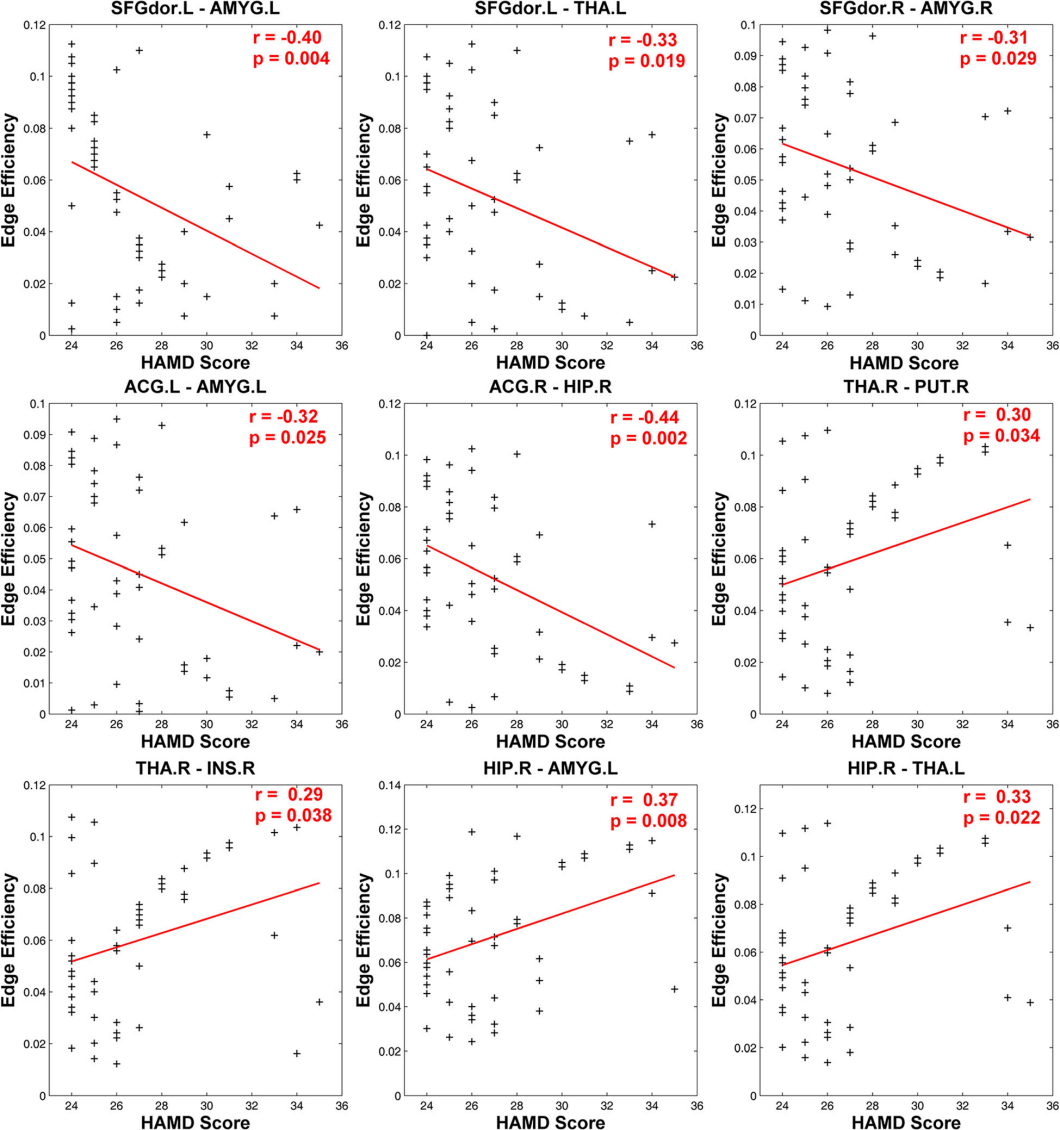
Upper scatter diagram shows correlation analysis between MDD edge efficiency and HAMD Scores. The figure shows that the edge efficiency between left dorsolateral prefrontal cortex and regions (*left amygdala, left thalamus*), right dorsolateral prefrontal cortex and regions (right amygdala, left anterior cingulate gyrus and right amygdala) has negative correlation with HAMD Scores. In addition, the edge efficiency between right thalamus and regions (*right putamen, right insula*), left hippocampus and regions (left amygdala, left thalamus) has positive correlation with HAMD Scores

In addition, we also calculated pearson correlation coefficients to examine how edge efficiency relate with clinical severity. Results suggested that HAMD score was negative correlated with edge efficiency which connected between cognitive control regions (dorsal superior frontal gyrus and anterior cingulate cortex) and affective processing regions (amygdala, thalamus and hippocampus). While HAMD score was found to positive correlate with edge efficiency which connected within affective processing regions (putamen, insula, hippocampus, amygdala and thalamus) (see Fig. 6).

## Discussion

In this paper, we investigated the node and edge efficiency of functional connectivity networks in MDD patients. The same analysis methods with different independent datasets and structural templates repeated the results. Results showed that MDD patients increased nodal efficiency in limbic regions which were associated with affective processing, yet decreased nodal efficiency in regions of cognitive control processing, such as DLPFC and ACC. However, MDD patients decreased in edge efficiency involving connectivity between regions of cognitive control processing systems. Our results demonstrated that the network efficiency of MDD patients are altered within cognitive control and affective processing systems.

However, there are several issues needed to be addressed. First, our results of increased nodal efficiency in limbic regions which were associated with affective processing, yet decreased nodal efficiency in regions of cognitive control processing, such as DLPFC and ACC in MDD patients were different from one fMRI study. Variations in clinical characteristics of MDD maybe one important factor to account for these discrepancies. Different clinical characteristics and depression severity may result in different brain activations patterns and further cause different brain network topological properties. Future studies, which use MDD patients with different clinical characteristics, may give us a more complete understanding of brain abnormalities. Secondly, the clinical diagnosis of MDD has some limitations and is easily affected by subjective knowledge and experience. Hence, the objective diagnosis of MDD has high clinical value in preventing severe disease. In addition, the present study suggested that the measurement of topological properties was a preferential candidate for diagnosing MDD. Thirdly, large studies have indicated that the cerebellum was closely associated with higher-order functions, including emotion regulation and cognitive processing, also have suggested that the cerebellum should be included in the pathophysiological models of MDD. However, because of the AAL template that we chose did not contain the cerebellum, the current study analyzed the functional brain network without cerebellum. In future, the cerebellum should be involved in the analyses by selecting the more comprehensive template or making a special analysis on the cerebellum. Finally, network disruptions in MDD patients is still in its preliminary stage, and there are still many issues to be deepening. Until now, MDD patients have been investigated widely using complex network theory. Next, we should forma hypothesis-generating framework to understand the relationships between abnormal topological properties and MDD.

In summary, the present study examined the node and edge properties of brain functional networks in MDD patients and normal controls using resting-state fMRI. Importantly, we replicated the results from independent datasets and then from same network analysis with different structural templates. Results demonstrated that network efficiency i.e., global efficiency, local efficiency, nodal efficiency and edge efficiency analysis revealed that depression patients showed small-world features, regions activation, the strength of connectivity in brain functional networks with MDD. These findings might provide effective potential biomarkers for clinic treatment of depression and further pathophysiological mechanisms of depressive patients.

First, in the present study, we validated the topological disorganization of depression using two independent datasets and different structural templates. It was well-known that, replicating promising findings in biomedicine was very difficult and replication rates might be about 25 % or less. However, in order to make results more reliable in the present study, we first used two independent datasets and then conducted the network topological properties analysis with different value of cost and structural templates. Previous studies [32] with topological disorganization of depression almost contained such limitations. Despite the differences between the subjects and templates examined in this study, the pattern of results was remarkably similar. Through the analysis, we reduced the false probability of result and increased the reliability of calculated result.

Then, we applied the nodal efficiency which indicates the importance of node in the whole network. The abnormal nodal efficiency in MDD compared to controls in regions was obvious. The increases in nodal efficiency occur in regions involving amygdale, thalamus, fusiform gyrus, hippocampus, rectus gyrus and middle temporal gyrus. As previous studies suggested, these regions were core parts of affective processing network, which exhibited dysfunction in MDD. Specifically, amygdala would show greater reactivity and longer lasting when depressed patients process negative stimuli. Hippocampus would show enhanced activity in recall of negative, not positive stimuli after encoding in the amygdala [7, 14]. Our study further shed light on the abnormality of the affective processing system of MDD from the perspective of network. In addition, the decrease in nodal efficiency is also found in MDD in the present study, involving DLPFC and ACC. Decreased activation in regions of executive control system has also been reported in some other studies [9, 12, 14]. Prior studies investigating inhibition control on negative stimuli in MDD, which suggested that depressed patients need greater cognitive effort to divert attention away from negative stimuli [10, 11, 24]. Being consistent with prior findings, MDD patients in the present study showed less effective cognitive control compared with normal subjects. Moreover, the present study highlighted the role of DLPFC and ACC in affective control and in MDD. To further verify the differences in nodal efficiency of regions in MDD patients and normal controls, we made correlation analysis between nodal efficiency and HAMD scores. Our results demonstrated negative correlation between the decrease in MDD patients’ nodal efficiency and HAMD scores, but positive correlation between the increase of nodal efficiency and HAMD scores. These findings indicated that with the growing severity of depression, the ability of cognitive control in MDD decreased while dysfunction of affective processing increased. In sum, increases in nodal efficiency in regions of affective processing and decreases in regions of cognitive control suggested that impaired cognitive control combined with abnormal affective processing lead to development of MDD.

More interestingly, we found some decreased edge efficiency, i.e., connectivity between DLPFC and limbic regions (amygdala, thalamus, hippocampus and palladium) and connectivity between ACC and limbic regions (hippocampus, amygdala, putamen). On the other hand, some increased edge efficiency, i.e., connectivity between thalamus and regions (amygdala, putamen, insula) and connectivity between hippocampus and regions (amygdala, thalamus) was found. These alterations in edge efficiency are compatible with previous MDD studies [2, 21, 30]. The increased connectivity between regions in the limbic system might indicate that bottom-up affective processing was excessively processed in MDD [7, 27]. What’s more, the decreased connectivity between regions of cognitive control (DLPFC and ACC) and regions in limbic system might indicate that top-down cognitive control of MDD has less effect upon affective processing [7, 12]. To further verify the differences in connectivity between regions in MDD patients and normal subjects, we made correlation analysis between edge efficiency and HAMD scores. As expected, the result suggested that the decrease in MDD patients’ edge efficiency (top-down control on affective processing) has negative correlation with HAMD scores, while the increase in edge efficiency (bottom-up affective processing) had positive correlation with HAMD scores. These correlation analysis results confirmed our previous results.

## Conclusions

Our results show that MDD obvious increase in nodal efficiency in some regions (i.e., amygdala, thalamus, fusiform gyrus, hippocampus, rectus gyrus and middle frontal gyrus regions) and significant decrease in nodal efficiency was also found in regions in MDD (i.e., DLPFC and ACC). At the same time. The results indicate that obvious decrease in edge efficiency of MDD groups between DLPFC and regions (amygdala, thalamus, hippocampus, palladium), ACC and regions (amygdala, middle frontal gyrus, hippocampus, putamen). In addition, we also found significant increase in edge efficiency of MDD groups between thalamus and regions (amygdala, putamen, insula), between hippocampus and regions (amygdala, thalamus). The findings might shed light on the pathological mechanism of depression and provide potential biomarkers for clinic treatment of depression.

## Additional file

Additional file 1: Disrupted functional connectivity networks in depression. (DOCX 37 kb)

## Abbreviations

ACC: Anterior cingulate cortex
DLPFC: Dorsolateral prefrontal cortex
HAM-D: Hamilton Depression Rating Scale
MDD: Major depressive disorder
