# Supplementary material for: Altered network efficiency in major depressive disorder
Source: BMC Psychiatry. 2016 Dec 17;16:450. doi: 10.1186/s12888-016-1053-9 (PMC5164918; doi:10.1186/s12888-016-1053-9)
Supplement: Additional file 1: — Disrupted functional connectivity networks in depression. (DOCX 37 kb) [file 12888_2016_1053_MOESM1_ESM.docx]

**Disrupted Functional Connectivity Networks in**

**Major Depressive Disorder—replicated for two independent datasets**

**Supplement 1**

**Supplemental Result**

In order to verify the reliability of the results, we calculated the result with different value of cost and with Harvard-Oxford Atlases template. In addition, we also tested the result in Dataset 2.

Result replicated at the cost of 0.17 and 0.28 and within Harvard-Oxford Atlases template (see table_s1 to S8). Results suggested that MDD patients demonstrated obvious increase in nodal efficiency in some affective processing regions (i.e., amygdale, thalamus, hippocampus, caudate nucleus) and significant decrease in nodal efficiency is also found in cognitive control regions in MDD (i.e., DLPFC and ACC). Results about edge efficiency showed that MDD group demonstrated significant decrease in regions between DLPFC and regions (amygdala, hippocampus), ACC and regions (amygdala, thalamus). In addition, we also found significant increase in edge efficiency of MDD group between thalamus and regions (amygdala, putamen), between hippocampus and regions (amygdala, thalamus).

In dataset 2 (30 MDD and 36 controls), the results were still replicated (see table_s9 to s14). First, MDD group demonstrated significant increase in nodal efficiency in some affective processing regions and decrease in nodal efficiency in cognitive control regions (i.e., DLPFC and ACC) at the cost of 0.23 with two structural templates (see table_s10 and s13). In addition, MDD group showed significant decrease of edge efficiency between DLPFC ( and ACC) and affective processing related regions (amygdala, hippocampus thalamus). Moreover, we also found significant increase in edge efficiency of MDD group between thalamus and regions (amygdala, putamen), between hippocampus and regions (amygdala, thalamus) (see table_s11 and s14).

Table_S1. The results of two-samples test in local efficiency with AAL template (Dataset 1)

| **Cost** | **t Value** | **P Value** |
| --- | --- | --- |
| 0.15 | 2.06 | 0.042 |
| 0.16 | 2.05 | 0.043 |
| 0.17 | 2.15 | 0.034 |
| 0.18 | 2.20 | 0.030 |
| 0.19 | 2.47 | 0.015 |
| 0.20 | 2.69 | 0.009 |
| 0.21 | 3.91 | <0.001 |
| 0.22 | 2.44 | 0.016 |
| 0.23 | 2.08 | 0.040 |
| 0.24 | 2.04 | 0.044 |
| 0.25 | 2.14 | 0.035 |
| 0.26 | 2.16 | 0.033 |
| 0.27 | 2.14 | 0.035 |
| 0.28 | 2.18 | 0.032 |
| 0.29 | 2.04 | 0.044 |
| 0.30 | 2.00 | 0.048 |

Table_S2. The results of two-sample test in nodal efficiency at cost of 0.17 with AAL template (Dataset 1)

| **Regions** | **Hemisphere** | **t value(p value)** | **r value(p value)** |
| --- | --- | --- | --- |
| **Depression<Control** | | | |
| Superior frontal gyrus, dorsolateral | L | -2.12(0.037) | -0.45(0.011) |
|  | R | -2.01(0.048) | -0.32(0.025) |
| Anterior cingulate gyrus | L | -2.70(0.008) | -0.42(0.003) |
|  | R | -2.40(0.018) | NS |
| **Depression>Control** | | | |
| Hippocampus | L | 2.54(0.013) | 0.31(0.030) |
|  | R | 2.21(0.029) | 0.44(0.001) |
| Amygdala | L | 2.06(0.042) | NS |
|  | R | 2.56(0.012) | 0.30(0.038) |
| Caudate nucleus | L | 2.90(0.005) | 0.32(0.023) |
| Thalamus | L | 2.27(0.026) | NS |
|  | R | 2.15(0.034) | NS |

Table_S3. The results of two-sample test in edge efficiency at cost of 0.17 with AAL template (Dataset 1)

| **Regions of seed** | **Connected regions** | **t value(p value)** | **r value(p value)** |
| --- | --- | --- | --- |
| **Depression<Control** | | | |
| Left Superior frontal gyrus, dorsolateral | Right Amygdala | -2.02(0.047) | -0.29(0.042) |
|  | Left Hippocampus | -2.28(0.025) | NS |
| Left Anterior cingulate gyrus | Left Amygdala | -2.13(0.036) | -0.44(0.002) |
| Right Anterior cingulate gyrus | Right Amygdala | -2.09(0.040) | -0.32(0.025) |
|  | Left Thalamus | -2.33(0.022) | NS |
| **Depression>Control** | | | |
| Right Thalamus | Right Amygdala | 3.02(0.003) | NS |
|  | Right Putamen | 2.34(0.021) | NS |
| Right Hippocampus | Left Amygdala | 2.44(0.016) | 0.33(0.019) |
|  | Left Thalamus | 2.11(0.037) | 0.32(0.025) |

Table_S4. The results of two-sample test in nodal efficiency at cost of 0.28 with AAL template (Dataset 1)

| **Regions** | **Hemisphere** | **t value(p value)** | **r value(p value)** |
| --- | --- | --- | --- |
| **Depression<Control** | | | |
| Superior frontal gyrus, dorsolateral | R | -2.14(0.035) | -0.43(0.002) |
| Middle frontal gyrus | L | -2.18(0.032) | -0.35(0.013) |
|  | R | -2.50(0.014) | NS |
| Anterior cingulate gyrus | L | -2.21(0.029) | -0.34(0.018) |
|  | R | -2.55(0.012) | -0.33(0.022) |
| **Depression>Control** | | | |
| Hippocampus | L | 2.12(0.036) | NS |
| Amygdala | L | 2.24(0.027) | 0.30(0.036) |
|  | R | 2.55(0.013) | 0.32(0.028) |
| Pallidum | L | 2.56(0.012) | NS |
|  | R | 2.39(0.019) | NS |
| Thalamus | L | 2.37(0.020) | 0.33(0.022) |
|  | R | 2.56(0.012) | 0.41(0.003) |
| Middle temporal gyrus | R | 2.19(0.031) | NS |

Table_S5. The results of two-sample test in edge efficiency at cost of 0.28 with AAL template (Dataset 1)

| **Regions of seed** | **Connected regions** | **t value(p value)** | **r value(p value)** |
| --- | --- | --- | --- |
| **Depression<Control** | | | |
| Left Anterior cingulate gyrus | Left Amygdala | -2.51(0.014) | NS |
|  | Right Middle temporal gyrus | -2.34(0.021) | -0.33(0.019) |
|  | Right Caudate nucleus | -2.67(0.009) | -0.32(0.026) |
| Right Anterior cingulate gyrus | Right Hippocampus | -2.40(0.019) | -0.37(0.008) |
|  | Right pallidum | -2.49(0.015) | NS |
| **Depression>Control** | | | |
| Right Thalamus | Left putamen | 2.71(0.008) | 0.40(0.005) |
|  | Right Insula | 2.42(0.018) | 0.30(0.037) |
| Left Hippocampus | Left Amygdala | 2.69(0.009) | 0.36(0.010) |
|  | Right Amygdala | 2.21(0.029) | NS |

Table_S6. The results of two-sample test in local efficiency with Harvard-Oxford Atlases template (Dataset 1)

| **Cost** | **t Value** | **P Value** |
| --- | --- | --- |
| 0.17 | 2.27 | 0.026 |
| 0.18 | 2.49 | 0.015 |
| 0.19 | 2.24 | 0.027 |
| 0.20 | 2.83 | 0.006 |
| 0.21 | 3.96 | <0.001 |
| 0.22 | 2.53 | 0.013 |
| 0.23 | 2.23 | 0.028 |
| 0.24 | 2.24 | 0.028 |
| 0.25 | 2.60 | 0.011 |
| 0.26 | 2.25 | 0.027 |
| 0.27 | 2.53 | 0.013 |
| 0.28 | 2.07 | 0.041 |

Table_S7. The results of two-sample test in nodal efficiency at cost of 0.21 with Harvard-Oxford Atlases template (Dataset 1)

| **Regions** | **MNI coordinates** | | | **t value (p value)** | **r value (p value)** |
| --- | --- | --- | --- | --- | --- |
|  | x | y | z |  |  |
| **Depression<Control** | | | | | |
| Left superior frontal gyrus | -23 | 32 | 46 | -2.29(0.025) | -0.30(0.034) |
| Right superior frontal gyrus | 28 | 1 | 56 | -2.04(0.045) | -0.35(0.014) |
| Right middle frontal gyrus | 41 | 26 | 37 | -2.52(0.013) | NS |
| Left anterior cingulate gyrus | 1 | 35 | 23 | -3.24(0.002) | -0.33(0.019) |
| Right anterior cingulate gyrus | 6 | 45 | 5 | -2.29(0.024) | -0.33(0.023) |
| **Depression>Control** | | | | | |
| Left hippocampus | -32 | -19 | -19 | 2.45(0.016) | NS |
| Right amygdala | 20 | -10 | -17 | 2.99(0.004) | 0.31(0.032) |
| Left amygdala | -20 | -5 | -17 | 2.23(0.028) | NS |
| Right caudate | 14 | 16 | 6 | 2.51(0.014) | NS |
| Left putamen | -29 | 3 | 3 | 2.46(0.016) | 0.37(0.009) |
| Right Putamen | 26 | 3 | -1 | 2.86(0.005) | 0.36(0.011) |
| Left thalamus | -14 | -30 | 0 | 2.09(0.039) | NS |
| Left thalamus | -9 | -16 | 9 | 2.07(0.041) | NS |
| Right thalamus | 11 | -18 | 8 | 2.64(0.010) | NS |
| Right hippocampus | 18 | -32 | -3 | 2.54(0.013) | 0.29(0.042) |

Table_S8. The results of two-sample test in edge efficiency at cost of 0.21 with Harvard-Oxford Atlases template (Dataset 1)

| **Region of seed** | **Coordinates** | | |  | **Connected region** | **MNI coordinates** | | | **t value (p value)** | **r value (p value)** |
| --- | --- | --- | --- | --- | --- | --- | --- | --- | --- | --- |
|  | x | y | z |  |  | x | y | z |  |  |
| **Depression<Control** | | | | | | | | | | |
| Right middle frontal gyrus | 41 | 26 | 37 |  | Left hippocampus | -32 | -19 | -19 | -2.34(0.022) | NS |
|  |  |  |  |  | Right caudate | 14 | 16 | 6 | -2.78(0.007) | -0.32(0.026) |
| Left ACC | 1 | 35 | 23 |  | Left amygdala | -20 | -5 | -17 | -2.05(0.043) | -0.36(0.011) |
| Right ACC | 6 | 45 | 5 |  | Left hippocampus | -32 | -19 | -19 | -2.32(0.023) | -0.39(0.006) |
|  |  |  |  |  | Left putamen | -29 | 3 | 3 | -2.54(0.013) | NS |
| **Depression>Control** | | | | | | | | | | |
| Left thalamus | -9 | -16 | 9 |  | Left amygdala | -20 | -5 | -17 | 3.03(0.003) | 0.31(0.033) |
|  |  |  |  |  | Right hippocampus | 20 | -10 | -17 | 2.23(0.028) | NS |
|  |  |  |  |  | Right caudate | 14 | 16 | 6 | 2.05(0.043) | NS |
| Left hippocampus | -32 | -19 | -19 |  | Left amygdala | -20 | -5 | -17 | 2.43(0.017) | 0.35(0.013) |
|  |  |  |  |  | Right insula | 40 | -9 | -5 | 2.38(0.020) | NS |

ACC = anterior cingulate gyrus

Table_S9. The results of two-sample test in local efficiency with AAL template (Dataset 2)

| **Cost** | **t Value** | **P Value** |
| --- | --- | --- |
| 0.17 | 2.25 | 0.029 |
| 0.18 | 2.50 | 0.015 |
| 0.19 | 2.46 | 0.017 |
| 0.20 | 2.29 | 0.025 |
| 0.21 | 2.51 | 0.015 |
| 0.22 | 2.78 | 0.007 |
| 0.23 | 3.15 | 0.003 |
| 0.24 | 2.62 | 0.011 |
| 0.25 | 2.78 | 0.007 |
| 0.26 | 2.13 | 0.037 |
| 0.27 | 2.45 | 0.017 |

Table_S10. The results of two-sample test in nodal efficiency at the cost of 0.23 with AAL template ( Dataset 2)

| **Regions** | **Hemisphere** | **t value(p value)** | **r value(p value)** |
| --- | --- | --- | --- |
| **Depression<Control** |  |  |  |
| Superior frontal gyrus,dorsolateral | L | -2.84(0.006) | -0.39(0.019) |
| Superior frontal gyrus,orbital part | L | -2.55(0.013) | -0.38(0.023) |
|  | R | -3.36(0.001) | NS |
| Anterior cingulate gyrus | L | -2.78(0.007) | -0.45(0.006) |
|  | R | -2.28(0.026) | -0.49(0.002) |
| **Depression>Control** |  |  |  |
| Hippocampus | L | 2.13(0.036) | NS |
|  | R | 2.74(0.008) | 0.34(0.040) |
| Amygdala | L | 2.09(0.040) | 0.37(0.027) |
|  | R | 2.64(0.010) | 0.41(0.012) |
| Thalamus | L | 2.50(0.015) | NS |
|  | R | 2.25(0.028) | NS |

Table_S11. The results of two-sample test in edge efficiency at the cost of 0.23 with AAL template ( Dataset 2)

| **Region of seed** | **Connected regions** | **t value(p value)** | **r value(p value)** |
| --- | --- | --- | --- |
| **Depression<Control** | | | |
| Left Superior frontal gyrus, dorsolateral | Left Amygdala | -2.24(0.028) | NS |
|  | Right Amygdala | -3.12(0.003) | -0.38(0.021) |
| Left Anterior cingulate gyrus | Left Amygdala | -2.64(0.010) | -0.48(0.003) |
|  | Right Middle temporal gyrus | -2.54(0.013) | -0.43(0.009) |
| Right Anterior cingulate gyrus | Right Amygdala | -2.70(0.009) | -0.40(0.016) |
|  | Left Thalamus | -2.46(0.017) | NS |
| **Depression>Control** | | | |
| Left Hippocampus | Left Amygdala | 2.79(0.007) | NS |
|  | Left Thalamus | 2.30(0.024) | 0.36(0.033) |
| Right Thalamus | Right Amygdala | 2.05(0.044) | 0.44(0.008) |

Table_S12. The results of two-sample test in local efficiency with Harvard-Oxford Atlases template (Dataset 2)

| **Cost** | **t Value** | **P Value** |
| --- | --- | --- |
| 0.16 | 2.05 | 0.045 |
| 0.17 | 2.21 | 0.031 |
| 0.18 | 2.54 | 0.014 |
| 0.19 | 2.54 | 0.014 |
| 0.20 | 2.15 | 0.036 |
| 0.21 | 2.79 | 0.007 |
| 0.22 | 2.90 | 0.005 |
| 0.23 | 3.61 | <0.001 |
| 0.24 | 2.82 | 0.007 |
| 0.25 | 2.29 | 0.026 |
| 0.26 | 2.22 | 0.031 |
| 0.27 | 2.15 | 0.036 |
| 0.28 | 2.08 | 0.042 |

Table_S13. The results of two-samples test in nodal efficiency at cost of 0.23 with Harvard-Oxford Atlases template (Dataset 2)

| **Regions** | **MNI coordinates** | | | **t value(p value)** | **r value(p value)** |
| --- | --- | --- | --- | --- | --- |
|  | **x** | **y** | **z** |  |  |
| **Depression<Control** | | | | | |
| Left superior frontal gyrus | -23 | 32 | 46 | -2.43(0.018) | -0.49(0.002) |
| Right superior frontal gyrus | 28 | 1 | 56 | -2.88(0.005) | -0.38(0.022) |
| Right frontal pole | 1 | 58 | -8 | -2.54(0.013) | NS |
| Right frontal pole | 27 | 61 | -3 | -2.37(0.021) | NS |
| Left anterior cingulate gyrus | 1 | 35 | 23 | -2.25(0.028) | -0.34(0.042) |
| Right anterior cingulate gyrus | 6 | 45 | 5 | -2.92(0.005) | -0.36(0.029) |
| **Depression>Control** | | | | | |
| Left hippocampus | -32 | 19 | -19 | 2.42(0.018) | 0.45(0.006) |
| Right amygdala | 20 | -10 | -17 | 2.25(0.028) | 0.38(0.022) |
| Left amygdala | -20 | -5 | -17 | 2.03(0.046) | 0.34(0.044) |
| Right caudate | 14 | 16 | 6 | 2.67(0.009) | 0.44(0.007) |
| Left thalamus | -14 | -30 | 0 | 2.52(0.014) | NS |
| Left thalamus | -9 | -16 | 9 | 2.87(0.005) | NS |
| Right thalamus | 11 | -18 | 8 | 2.05(0.044) | 0.35(0.039) |
| Right hippocampus | 18 | -32 | -3 | 2.61(0.011) | NS |

Table_S14. The results of two-samples test in edge efficiency at cost of 0.23 with Harvard-Oxford Atlases template (Dataset 2)

| **Region of seed** | **Coordinates** | | |  | **Connected region** | **MNI Coordinates** | | | **t value(p value)** | **r value(p value)** |
| --- | --- | --- | --- | --- | --- | --- | --- | --- | --- | --- |
|  | **x** | **y** | **z** |  |  | **x** | **y** | **z** |  |  |
| **Depression<Control** | | | | | | | | | | |
| Left superior frontal gyrus | -23 | 32 | 46 |  | Left amygdala | -20 | -5 | -17 | -2.31(0.024) | -0.52(0.001) |
|  |  |  |  |  | Right fusiform gyrus | 27 | -48 | -14 | -2.85(0.006) | -0.39(0.020) |
| Right frontal pole | 27 | 61 | -3 |  | Right hippocampus | 20 | -10 | -17 | -2.41(0.019) | NS |
| Left ACC | 1 | 35 | 23 |  | Right hippocampus | 20 | -10 | -17 | -2.01(0.048) | NS |
|  |  |  |  |  | Right caudate | 14 | 16 | 6 | -2.58(0.012) | -0.40(0.015) |
| Right ACC | 6 | 45 | 5 |  | Left thalamus | -14 | -30 | 0 | -3.27(0.002) | -0.37(0.027) |
|  |  |  |  |  | Left thalamus | -9 | -16 | 9 | -3.09(0.003) | NS |
| **Depression>Control** | | | | | | | | | | |
| Left hippocampus | -32 | -19 | -19 |  | Left amygdala | -20 | -5 | -17 | 2.51(0.015) | 0.36(0.032) |
|  |  |  |  |  | Right thalamus | 11 | -18 | 8 | 2.36(0.021) | 0.44(0.007) |
| Left amygdala | -20 | -5 | -17 |  | Left thalamus | -14 | -30 | 0 | 3.11(0.003) | NS |
|  |  |  |  |  | Left thalamus | -9 | -16 | 9 | 3.07(0.003) | NS |

ACC= anterior cingulate gyrus
